# Responsive Hydrogels for Label-Free Signal Transduction within Biosensors

**DOI:** 10.3390/s100504381

**Published:** 2010-04-30

**Authors:** Kamila Gawel, David Barriet, Marit Sletmoen, Bjørn Torger Stokke

**Affiliations:** 1 Biophysics and Medical Technology, Department of Physics, The Norwegian University of Science and Technology, NTNU NO-7491 Trondheim, Norway; E-Mails: kamila.gawel@ntnu.no (K.G.); david.barriet@ntnu.no (D.B.); marit.sletmoen@ntnu.no (M.S.); 2 NTNU NanoLab, The Norwegian University of Science and Technology, NTNU NO-7491 Trondheim, Norway

**Keywords:** biospecific hydrogels, biosensors

## Abstract

Hydrogels have found wide application in biosensors due to their versatile nature. This family of materials is applied in biosensing either to increase the loading capacity compared to two-dimensional surfaces, or to support biospecific hydrogel swelling occurring subsequent to specific recognition of an analyte. This review focuses on various principles underpinning the design of biospecific hydrogels acting through various molecular mechanisms in transducing the recognition event of label-free analytes. Towards this end, we describe several promising hydrogel systems that when combined with the appropriate readout platform and quantitative approach could lead to future real-life applications.

## 1. Introduction

Hydrogel materials have found widespread biomedical applications in regenerative medicine and drug delivery. There is also an increasing interest in hydrogels within biosensing. While biosensing in general takes advantage of various readout platforms based on electrochemical, mechanical or optical detection principles (e.g., amperometric, surface plasmons, fluorescence, dual polarization interferometry and others), hydrogel specific properties appear to be less exploited in biosensing applications. A biosensor can be viewed as a combination of a selective detection/recognition unit, a transducing unit and a readout part. The detection unit is designed to react in the presence of the desired analyte. The function of the transducing part of the biosensor is to convert the presence of the relevant analyte into an output that can readily be measured by the actual output system. These functionalities need to be optimized in a holistic design, *i.e.*, not all combinations of primary detection units and readout technologies will perform equally well. Within the field of biosensors, hydrogels have been applied for two main purposes: increase the loading capacity of an analyte by transforming a conventional 2D immobilization scheme into a 3D meshwork or to take advantage of hydrogel specific properties (swelling, phase transitions and properties that derive from them). In the latter, the design of the matrix design supports signal transduction by altering the degree of hydrogel swelling associated with the specific recognition/detection of the analyte.

The plethora of responsive characteristics displayed by various hydrogels includes changes of equilibrium swelling volume due to changes in e.g., parameters such as solvent pH [1,2] temperature [3,4], ionic strength [5,6], electric fields [7], and surfactants [8], among others. Such types of responses are usually not sufficiently specific for the hydrogel materials to be applied as specific signal transduction materials within biosensors. The implementation of a biospecific hydrogel response can be designed to utilize different molecular mechanisms, and thus different parameters in the Flory-Rehner-Donnan theory of hydrogel swelling, as herein indicated. The theory is based on random-mixing lattice model, assumes Gaussian distribution of the polymer chains and neglects the electrostatic interaction between charges present in the network. Even though there are several reviews within bioresponsive hydrogels [9–13], a linkage between molecular mechanisms governing the response and the hydrogel swelling theory appear not to be widely explored. Briefly, in a first approximation, the equilibrium state of a polyelectrolyte hydrogel is described by total zero osmotic pressure (Π) of the gel. The osmotic pressure of an ionic hydrogel is in the simplest form of the theory, assumed to consist of three additive contributions arising from different molecular mechanisms: first, the free energy contribution arising from the mixing of the polymer with the solvent, Π_mix_; second, the elastic retractive force associated with deformation of the polymer chains, Π_el_; and third, the difference in mobile ions concentration inside and outside of the gel, Π_ion_ (if the polymer chains are charged). The total osmotic pressure can then be written as [6,14–17]:

(1) $$\Pi_{\mathrm{mix}}+\Pi_{\mathrm{el}}+\Pi_{\mathrm{ion}}=\frac{\mathrm{RT}}{V_{1}}\left(\text{ln}\varphi_{1}+\varphi_{2}+\chi \varphi_{2}^{2}\right)+\frac{\upsilon \mathrm{RT}}{V_{0}}\left(\frac{\varphi_{2}}{{2\varphi }_{2,0}}-{\left(\frac{\varphi_{2}}{\varphi_{2,0}}\right)}^{1/3}\right)+\mathrm{RT}\Delta C_{\mathrm{tot}}$$

In Equation 1, subscript 1 and 2 of the volume fractions φ denote the solvent and polymer phase respectively. V_1_ is the molar volume of the solvent, υ is the molar number of elastic active polymer chains in the gel at the reference volume fraction φ_2,0_, V_0_ is the gel volume for the reference state, R is the molar gas constant, T is the absolute temperature, and χ the Flory-Huggins interaction parameter taking into account the energy of interdispersing polymer and solvent molecules. The role of the Flory-Huggins parameter in the hydrogel swelling has been addressed in more detail elsewhere [13] and as the swelling mechanism involving changes of the parameter was utilized rather in the frame of thermo- than selectively bioresponsive hydrogels, it is not the focus here. The total difference in molar concentration of mobile ions between the gel and the surrounding aqueous solution, Δ*C_tot_*, is given by the Donnan equilibrium and theoretical expression including molecular parameters of the network and the valence of the electrolytes are described [6].

Within this framework, an understanding of the mechanism explaining how responsive gels adopt a new equilibrium swelling volume, 1/φ_2_, can be identified by considering the effect of the various parameters in Equation 1. For instance, bioresponsive hydrogels built by incorporation of an antigen-antibody pair as a physical crosslink that dissociate in the presence of its specific antigen/antibody, yields a swelling response that mainly originates from changes in the crosslink density, parameter υ in Equation 1. Alternatively, a bioresponsive hydrogel designed by immobilization of an enzyme that catalyzes the transformation of a substrate from its non-ionic form to its ionic form (e.g., glucose oxidase) will be primarily mediated by changes in the Δ*C_tot_* term in Equation 1.

Traditionally, the determination of hydrogel-swelling characteristics has been performed either optically, *i.e.*, by imaging a piece of gel using a light microscope, or by weighing following blotting off excess water. More accurate detection methods have been realized by incorporating the hydrogels into sensors such as conductimetric [18], liquid column length [19], or optical sensing [20–23]. Additionally, determination of changes in swelling using dynamic light scattering methods has been reported when the size of microgel particles was appropriate [24,25]. The precision of the detection of changes in hydrogel swelling volume limits the applicability of responsive gels within biosensors and as a fundamental tool for understanding of functional properties of responsive polymer materials. While biospecific recognition and signal transduction exploiting hydrogel properties may be beneficial, their introduction in a polymer network can inversely affect diffusion properties of the target analyte, thus offsetting the pre-cited advantages.

Within this framework, we will review the present status of hydrogel materials applications in biosensing. The scene is set by first performing some calculations based on further development of Equation 1 to allude to the effects of analyte induced changes in equilibrium swelling based on various mechanisms. Section 3 includes an overview of reported strategies followed by readout platforms needed to design a functional biosensor (Section 4) before the concluding remarks. We limit ourselves to label-free detection schemes [26,27], *i.e.*, that can be applied without prior labeling of the analyte. Such label-free detection schemes have the advantage to potentially support direct detection within bodily fluids.

## 2. Biospecific Response of Hydrogels – What Can Be Achieved?

Bioresponsive hydrogels can be realized where the changes in the equilibrium swelling is designed to primarily act by changing the cross-link density, the charge density of elastic chains of the network or pH of the aqueous phase of the network (see below). How large a change one can expect based on these various mechanisms will be alluded to by performing some calculations based on elaborations of Equation 1. To include the effect of finite extensibility of elastically active chains, the expression of Π*_el_* was modified using the inverse Langevin function L^−1^ [14]:

(2) $$\Pi_{\mathrm{el}}=\frac{\upsilon \mathrm{RT}}{V_{0}}\left(\frac{\lambda_{\text{max}}}{3}L^{-1}(\lambda /\lambda_{\text{max}}){\left(\frac{\varphi_{2}}{\varphi_{2,0}}\right)}^{1/3}-1\right)$$

where λ_max_, is the maximum linear extension ratio of the polymer chain. The actual extension is related to the swelling ratio through the relation *λ* = (*φ*_2,0_/*φ*_2_)^1/3^ for three-dimensional isotropic swelling.

**Figure 1.**
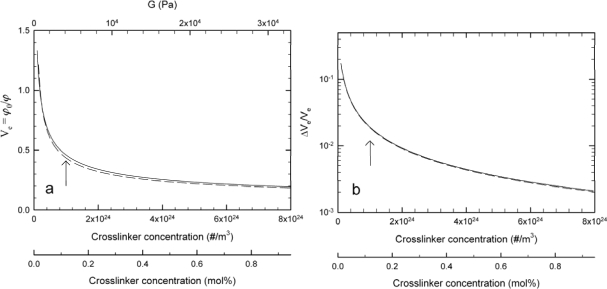
Example of simulation results showing the calculated equilibrium swelling ratio *versus* crosslink density (a) using Equation 2–5 for a 10% w/v hydrogel with charge density parameter equivalent molecular weight per charge (M_2_) of 2,500 g/mol e^−^ (continuous line) and uncharged polymer chains (broken line), Flory-Huggins interaction parameter χ = 0.46, maximum elastic chain stretching ratio λ_max_ = 15, and ionic strength of 0.1 M monovalent type salt added to the aqueous solution (only for the charged network case) and at a temperature of 25 °C. The network parameters are derived for a polyacrylamide based hydrogel and the crosslinker concentration is provided both in absolute numbers and mol% relative to the monomer of the polymer assuming a bis crosslinker. Additionally, an estimate of the shear storage modulus, G, over the range of hydrogel crosslinker densities are depicted (upper scale, Figure 1a). Figure 1b depicts the relative change in swelling volume *versus* crosslink density for the simulation depicted in Figure 1a. The arrows (Figure 1a,b) depict the crosslink density used in the calculations in Figure 2.

**Figure 2.**
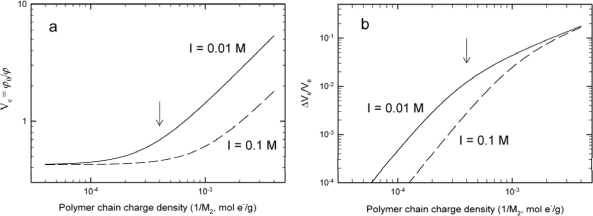
Example of simulation results showing the calculated equilibrium swelling ratio *versus* equivalent molecular weight per charge (M_2_) of the polymer network chains **(a)** using Equation 2–5 for a 10% w/v hydrogel with crosslinker concentration 1 × 10^24^ m^−3^, Flory Huggins interaction parameter χ = 0.46, maximum stretching ratio λ_max_ = 15, and ionic strength of 0.01 M and 0.1 M monovalent type salt added to the aqueous solution at a temperature of 25 °C. The crosslink concentration of 1 × 10^24^ m^−3^ correspond to 0.118 mol% of the crosslinker assuming the 10% w/v hydrogel is a polyacrylamide based hydrogel crosslinked with bis-acrylamide. Figure 2b depicts the relative change in swelling volume *versus* the equivalent molecular weight per charge of the polymer network chains for the simulation depicted in Figure 2a. The arrows (Figure 2a,b) depict the charge density (1/M_2_) used in the calculations in Figure 1.

Effects of analyte binding working through altered charge density of the elastic active chains are developed through the ionic term of Equation 1. The difference in electrolyte concentration inside and outside the gel is given by:

(3) $$\Delta C_{\mathrm{tot}}=(C_{+}+C_{-})-({C^{\prime }}_{+}+{C^{\prime }}_{-})$$

where C_+_ and C_−_ are the concentrations of positive and negative ions inside the gel, respectively, and the primed parameters the equivalent outside the gel. The ion concentration inside the gel can be calculated assuming that the relation for the Donnan equilibrium holds [14]. Electroneutrality and equality of chemical potential across the gel/solvent boundary for every ionic species are the two requirements needed to be fulfilled in this approach. Therefore, for anionic polymers in 1:1, 1:2 and 2:1 electrolytes, the Donnan equilibrium can be described by the following set of equations [28]:

(4) $$\begin{array}{l} z_{+}C_{+}^{\prime }=z_{-}C_{-}^{\prime }\\ z_{+}C_{+}=z_{-}C_{-}+Z_{p}C_{p}\\ \gamma_{\pm }^{2}C_{+}C_{-}=\gamma_{\pm }^{\prime 2}C_{+}^{\prime }C_{-}^{\prime } \end{array}$$

where z_+_ and z_−_ are the absolute values of the valences of the mobile ions, *γ*_±_^2^ are the square of the mean activity coefficient of the salt inside and outside the gel, respectively, Z_p_ is the effective number of counterions per polymer repeating unit, and C_p_ is the molar concentration of the polymer repeating unit. The term Z_p_C_p_ represents the additional counterions required to neutralize the fixed charge on the polymer network. In a biospecific responsive material acting according to such a mechanism, Z_p_ will vary depending on the amount of specifically bound analyte, and may also be a function of the pH. Parameter C_p_ is dependent on the swelling ratio of the gel. The term Z_p_C_p_ in Equation 4 is therefore expressed as [6]:

(5) $$Z_{p}C_{p}={\rho \phi }_{2}/M_{2}$$

where *ρ* is the mass density of the dry polymer network and *M_2_* is the molar mass of polymer per unit charge. Figures 1 and 2 depict examples of numerical calculations obtained using Equation 1–5, and parameter values as described in the legends. The algorithm implemented had previously been used to fit the parameters of the swelling theory to experimental swelling data for polysaccharide hydrogels of various charge densities in an aqueous solution at different ionic strengths [29]. Figure 1 depicts an example showing the effect of changes in crosslink density on the equilibrium swelling of a weakly charged and uncharged 10% w/v hydrogel in an aqueous salt solution. The smaller the crosslinking density of a gel, the larger the relative change in the hydrogel swelling volume (Figure 1b) gets. At the ionic strength selected for the calculation of the swelling of the weakly charged network, the equilibrium swelling is predicted to closely resemble that of the uncharged network, and both types of network increases in swelling volume the lower the cross-link density. This indicates that the design of the most sensitive hydrogel materials to specific biological signals working mainly through altered crosslink density is achieved at the smaller crosslink density (as expected). Hydrogel designs working mainly through altered crosslink density can be evaluated using the total crosslink density υ consisting of a covalent part and biospecific part (υ = υ_cov_ + υ_biospecific_, by molar ratio). However, in numerous practical applications, the material’s mechanical integrity as well as other material dependent properties (e.g., reflectivity, optical density) also needs to be taken into account.

The calculations of the equilibrium swelling volume *versus* the charge density (1/M_2_) (Figure 2) illustrate the interplay between the swelling sensitivity, the polymer chain charge density and the solution’s ionic strength. For most conditions, the lower ionic strength in the analyte bath fosters a more sensitive change in the equilibrium swelling volume, but the relative difference between the charge density induced swelling sensitivity decreases with increasing charge density (*i.e.*, with decreasing M_2_ parameter).

This first approach does not include effects due to loose-ends; elastic chain length heterogeneities are not addressed either. Furthermore, hydrogels for biosensors application may also need to be immobilized to a surface that, in view of the proper modeling, would represent swelling under a constrained condition. Nevertheless, this first result seems to indicate that a more explicit application of main trends appears feasible for optimizing design conditions for bioresponsive hydrogels.

## 3. Biospecific Response of Hydrogels – How Can It Be Achieved in Practice?

Whether one should aim at designing a hydrogel-based transducer working mainly through the charge density or the crosslink density mechanism depends on the properties of the analyte, and how the recognition element is integrated into the network topology. In the following section, practical aspects related to the Flory-Rhener-Donnan theory in biosensing using hydrogels are addressed. The sensitivity of hydrogels to such biological molecules as DNA, antigens, proteins, and small molecules of high biological importance is exemplified.

### 3.1. Osmotically Induced Hydrogel Response

Bioresponsive hydrogel swelling based on the ionic contribution to the osmotic pressure can be achieved in two different ways. Direct binding of a charged analyte molecule to the hydrogel network mediated by a specific recognition group yields a direct change in the M_2_ parameter that subsequently leads to an altered osmotic pressure or equilibrium hydrogel swelling. Alternatively, an uncharged analyte can be converted by a moiety (e.g., enzyme) covalently coupled to the hydrogel matrix thus generating ionic species within the aqueous phase of the hydrogel.

**Figure 3.**
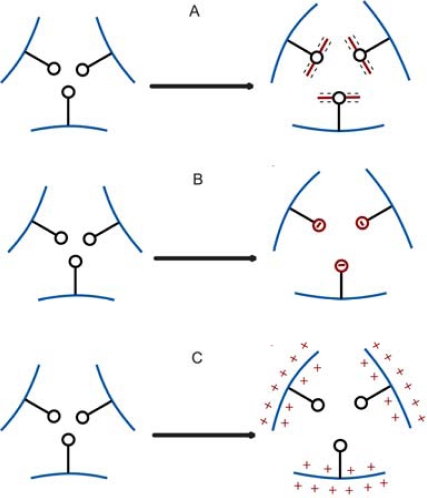
Schematic representation of osmotically-induced hydrogel swelling mechanisms: **(a)** binding of charged analyte molecules to the hydrogel binding sites [30], **(b)** generation of ionic species on the recognition element [33–35], **(c)** generation of ionic species on the sensitive polymer network [37–41].

In 1991 Tanaka and coworkers proved the concept of a hydrogel swelling due to the specific binding of charged molecules to the modified network [30]. Concanavalin A (ConA) is a lectin protein well known for its affinity to saccharides [31,32]. The lectin affinity depends on the type of saccharide. The authors physically entrapped ConA inside a poly-N-isopropylacrylamide network. Adding the anionic polysaccharide dextran sulfate resulted in gel swelling due to the affinity binding of dextran sulphate to ConA and a “charge entrapment” in the hydrogel (Figure 3a). Subsequent addition of nonionic saccharide a-methyl-d-mannopyranoside yielded a gel collapse due to its stronger affinity toward ConA than dextran sulphate. Due to the competitive binding of the neutral saccharide, the charged dextran was released and the osmotic pressure reduced so the polymer network shrunk to the relaxed state following diffusion of the released dextran.

The biological importance of glucose has motivated a large number of sensor developments. Direct binding of glucose to a polymer network does not induce swelling mediated by an ionic effect. However, Holtz and Asher [33–35] took advantage of the specific catalytic activity of an enzyme in a polymer hydrogel sensing principle for glucose, based on glucose oxidase conjugated to the gel. Glucose oxidase (GOx) oxidises b-d-glucose into d-glucono-1,5-lactone, which then hydrolyzes to gluconic acid. After glucose oxidation the enzyme is converted to the reduced anionic form (Equation 6). In the second stage GOx (red) is reoxid ized by molecular oxygen to its active form (Equation 7) [36].

(6) $$\text{GOx}\;(\text{ox})+\text{glucose}\to {\text{GOx}}^{-}(\text{red})+\text{gluconic acid}+H^{+}$$

(7) $${\text{GOx}}^{-}(\text{red})+H^{+}+O_{2}\to H_{2}O_{2}+\text{GOx}\;(\text{ox})$$

According to Holtz and Asher [33–35] the enzymatic action couples to the hydrogel swelling as follows. The formation of the reduced anionic species caused the glucose sensor swelling mediated by a change in the Donnan term (see Figure 3b). In the presence of oxygen the enzyme was regenerated. The gel remained swollen as long as glucose was present. However, in the deoxygenated solution, the hydrogel swelled and did not shrink over time. Under such anaerobic conditions the presence of glucose at the concentration of picomoles was detectable. The crystalline colloidal array polymerized within hydrogel and used as a readout platform (described in the next section) appears to support concentration detection in this range.

The above described oxidation mechanism accompanied with pH sensitivity of the hydrogel polymer was used by Ishihara *et al*. and Guiseppi-Elie *et al*. [37,38] to design a glucose responsive, insulin semipermeable material. The hydrogel polymer contained polyamines that became protonated at low pH. Enzymatic glucose oxidation to gluconic acid decreased the microenvironmental pH which resulted in amine protonation and hydrogel swelling (Figure 3c) and thus, enhanced the insulin permeability. Introduction of macroporosity into the glucose-sensitive and insulin-releasing hydrogel resulted in increased swelling degree and increased insulin permeability [39].

Hydrogel-based glucose sensing was also realized using phenylboronic acid as the primary recognition site [40]. Phenylboronic acid can form complexes with vicinal *cis* diols including carbohydrates. The dissociation constant of the acid decreased upon saccharide binding which resulted in the deprotonation of the molecule that subsequently affects the hydrogel swelling. However, the material also displayed altered swelling in the presence of other saccharides due to the primary recognition mechanism.

The pH-sensitive polymer motif was also applied to assay creatinine in bodily fluids. Creatinine is a product of creatine metabolism. The metabolite is filtered out of the blood by the kidneys. Creatinine level in a blood serum is an indicator of renal dysfunction [42]. To detect that level using hydrogel crystalline colloidal array, an enzyme (creatinine deiminase, CD) was used as the molecular recognition element [41]. The enzyme hydrolyzed creatinine to N-methylhydantoin with release of hydroxide anion (Equation 8) [43,44].

(8) $$\text{Creatinine}+H_{2}O\overset{\text{CD}}{\to }\text{N-Methylhydantion}+{\text{NH}}_{4}^{+}+{\text{OH}}^{-}$$

Following this scheme, the presence of creatinine caused an increase of pH inside the hydrogel. In addition to CD, 2-nitrophenol molecules were covalently attached to the network as pH sensors. The pH increase caused deprotonation of the phenol groups, which in turn gives rise to the hydrogel swelling. The hydrogel sensor was tested at pH∼7.4 and ionic strength ∼150 mM, both conditions close to those of bodily fluids. The response was reversible and reproducible in the range of creatinine concentrations 40–120 μM characteristic for human blood and plasma. At a concentration of creatinine of 300 μM the sensor required about 30 minutes to reach equilibrium.

### 3.2. Changes in Hydrogel Swelling Volume by Biospecific Changes in Crosslink Density

**Figure 4.**
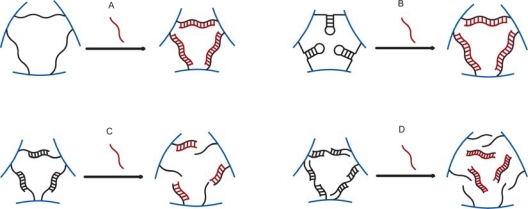
Schematics of DNA-crosslink designs and hydrogel swelling/shrinking mechanisms: **(a)** ssDNA with both ends anchored in the hydrogel network—shortening of the crosslinks upon binding of a complementary ssDNA probe [50], **(b)** ssDNA with intramolecular stem-loop base pairing with both ends anchored in the hydrogel network—elongation of the crosslinks upon binding of a complementary ssDNA probe [50], **(c)** two [51] and **(d)** three [49,52,53] partially complementary ssDNA—dissociation of the crosslinks upon complementary displacement in the presence of complementary ssDNA probe.

DNA sensitive hydrogels can be designed based on either a DNA supported increase or decrease in crosslink density, or changes in the equilibrium length of elastically active network chains. Recent technologies allow for the preparation of easily copolymerizable DNA strands into e.g., acrylamide based materials [45–47]. Oligonucleotide supported crosslink; either alone or in combination with bis-acrylamide, can be built with one or more oligonucleotides with capability for highly specific base-pair complementarity to neighboring strands. These crosslinks can be dissociated either by a change in temperature or by adding oligonucleotide with longer matching sequences through competitive replacement [48]. Moreover the length and the stability of the crosslinks can be engineered through the choice of the number of base pairs and base sequences [49], thus supporting a large number of different sequence recognition possibilities. Several network topologies including oligonucleotides can be implemented for preparation of analyte oliginucleotide sensitive hydrogel materials (e.g., Figure 4).

It has been reported that in the acrylamide hydrogels including two copolymerized oligonucleotides a third one, at least partially complementary to those copolymerized, supports a crosslink (Figure 4d). In such gel designs, the gel-sol transition can be induced either by a change of the temperature or by the addition of ssDNA with the proper base pair complementarity to the immobilized ones [49,52,53]. For the hydrogels with mixed hybridized DNA and covalent, non-dissociative crosslinks, swelling or shrinking of the hydrogel can be observed instead of sol-gel transition.

Murakami and Maeda reported on swelling and shrinking DNA responsive mechanisms for design (a) and (b) presented in Figure 4 [50]. Their acrylamide hydrogels contained either ssDNA or ssDNA with intramolecular loops base pairing as a cross-linker in addition to bis-acrylamide. They observed changes in hydrogel volume when exposed to complementary, single base-pair mismatch and uncomplementary ssDNA probes. They found that hydrogels containing ssDNA as elastic chains shrank in the presence of their complementary probes. Larger shrinkage and faster response was observed for long complementary sequences. Only slight shrinkage was observed for the uncomplementary probes due to the introduction of charged DNA molecules to the outer solution and the associated reduction of osmotic pressure. Even though hybridization leads to an increase of the charge density in the network which should manifest itself with the swelling of the gel, the dominant factor was the complementary base pairs crosslinking leading to shrinking of the gel. The reduced equilibrium swelling volume was mediated by reduction of base pair rise along the chain associated with the specific hybridization. Hydrogel designs with ssDNA loop structures, on the other hand, swelled when exposed to complementary probes. The effect was faster and more significant for the longest base pairs overlap. Non-complementary oligonucleotides did not induce such a swelling, and the response was also sensitive to single base pair mismatch.

Results comparable to those reported by Murakami and Maeda were obtained with a design incorporating two, at least partially hybridized, oligonucleotides into a hydrogel network as crosslinking junctions (Figure 4c) [51]. These hybrid hydrogels swelled when exposed to oligonucleotide probes with the number of base pair match exceeding that in the initial junction. The authors were able to detect the presence of an oligonucleotide probe at a concentration of 500 nanomoles. However, by extrapolating the curve optical path length change *versus* oligonucleotide concentration to the resolution of the fiber optic readout platform (2 nm), we estimated a sensitivity below 10 nanomolar [23,51]. The response rate increased with the probe concentration and the number of base pairs complementary to the junction oligonucleotide. Even though, the probe hybridization increased the network charge density, the major factor appeared to be related to the decrease of crosslinks density due to the competitive displacement of junction oligonucleotides. It was suggested that neither displacement hybridization kinetics nor diffusion rates of oligonucleotides were limiting steps but rather steric constraints within the hydrogel network.

The mechanism involving shortening of the crosslinks upon analyte binding was also used in the work of Yuan *et al*. [54]. Their hybrid hydrogels contained adenylate kinase3 (AK) involved in the crosslinks formation. AK is an enzyme which undergoes conformational changes following the binding of ATP molecule. In such modified hydrogel, the conformational changes of AK enzyme in the presence of ATP, were transformed into macroscopic hydrogel volume changes. The gel shrunk in the presence of ATP.

**Figure 5.**
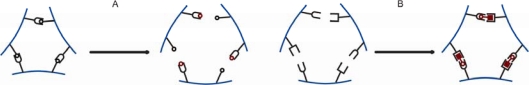
Schematic representations of swelling or shrinking mechanisms for the antigen sensitive hydrogels **(a)** antigen-antibody crosslinks dissociating in the presence of free antigen [55–57], **(b)** lectin-glycoprotein-antibody crosslinks formation in the presence of glycoprotein [58].

Miayata and coworkers have utilized semi-interpenetrating networks to design reversible antigen-responsive hydrogels [55–57]. Antigen-antibody mediated crosslinks between interpenetrating networks were realized by grafting rabbit IgG (AG) and goat anti-rabbit IgG (AB) as the antigen and the antibody, respectively. The crosslinks dissociated in the presence of a free native rabbit IgG, resulting in hydrogel swelling (Figure 5a). The phenomenon was explained in terms of differences in the binding constants of the native and the polymerized antigen. The crosslinks being intact in the presence of goat IgG indicated the high specificity of the hydrogel response. Swelling in the system was reversible in response to the changes in the antigen concentration. However, the reversibility was lost in case of antigen-antibody hydrogel without semi-interpenetrating structures.

The same authors applied the concept of biomolecular imprinting to the dynamic recognition of tumor-specific marker glycoprotein by lectin and antibody ligands [58]. Lectin-concanavalin A (Con A) was used as a ligand binding motif to the saccharide chain of α-fetoprotein (glycoprotein) and a polyclonal antibody was bound to the peptide unit. Both ligands were conjugated to the semi-interpenetrating hydrogel networks in the presence of the template glycoprotein so that the ligands formed a complex. After synthesis, the template glycoprotein was removed. This enabled formation of glycoprotein recognition sites based on the molecular imprinting principles. The response of imprinted and non-imprinted gels to target α-fetoprotein was different and showed the following features. While a slight swelling was observed for the non-imprinted gel, the imprinted gel shrunk. The shrinkage was due to the formation of a complex between bioconjugated Con A, antibody and the target glycoprotein (Figure 5b), thus yielding an increased cross-link density. Selectivity test employing ovalbumin, which has a saccharide chain similar to α-fetoprotein but different peptide units, showed no hydrogel shrinkage, thus indicating a highly selective material towards the tumor marker glycoprotein.

Hu *et al*. reported on combining molecular imprinting with photonic crystal readout platform within hydrogels. Their imprinted natural amino acid L-3,4-dihydroksyfenyloalanina (L-dopa) [59] within methacrylic acid hydrogel also supported chiral selectivity. L-dopa is able to form reversible complex with methacrylic acid by noncovalent interactions. The imprinted gel was found to be sensitive to L-dopa, but did not respond to its enantiomer, D-dopa. The observed shrinkage in the presence of a single enantiomer, within a concentration range of 10 nM to 10 mM, was suggested to arise from the formation of crosslinks.

The crosslink mechanism comparable to that used for glycoprotein recognition was applied to an antibiotic-sensing for the trigger-inducible release of human vascular endothelial growth factor [60]. The authors coupled genetically engineered bacterial gyrase subunit B (GyrB) to polyacrylamide polymer. GyrB was dimerized by the addition of the aminocoumarin antibiotic coumermycin. This resulted in the hydrogel formation. Subsequent addition of novobiocin (Albamycin) caused dissociation of the GyrB crosslinkages, resulting in gel-sol transition and liberation of the entrapped protein pharmaceutical

Much attention has been paid in the literature to glucose sensitive hydrogels. In addition to design strategies involving glucose oxidase enzyme and readout based on the osmotic pressure, as described above, hydrogels presenting glucose sensitive crosslink density have been demonstrated. Lectin protein—concanavalin A and phenylboronic acid supported glucose sensitive crosslinks incorporated into hydrogels are two viable routes.

The work by Brownlee *et al.* [32,61] and Kim *et al*. [62–64] on glucose-controlled insulin delivery systems based on ConA and glycosylated insulin have opened new research areas also within responsive hydrogels. Park *et al.* synthesized vinylpyrrolidinone-allylglucose and acrylamide-allylglucose copolymers and studied glucose-sensitive sol-gel transition in the systems containing ConA [65,66]. The formation of the hydrogel was due to the specific interaction between lectin receptor sites and glucose pendant groups of the polymeric chain. In the presence of free glucose, the opposite process: the peptization of a hydrogel, occurred. The concentration of the glucose at which gel-sol transition took place was about four times higher than the concentration of the monomers containing glucose. The gel could be recovered by glucose dialysis. The authors also studied insulin and lysozyme diffusion through a glucose-sensitive membrane containing ConA [67]. The release rate was dependent upon free glucose concentration.

Miyata *et al.* have copolymerized 2-glucosyloxyethyl methacrylate in the presence of a traditional crosslinker and ConA to obtain glucose-sensitive hydrogels [68]. Their observations revealed a direct correlation between the concentration of entrapped ConA and the crosslinking density, and an inverse correlation between the concentration of entrapped ConA and the swelling ratio of the hydrogel. The results suggest that Con A acted as an additional crosslinking point. The swelling ratio of the hydrogel increased in response to the presence of free glucose. Selectivity to glucose was not demonstrated in this system either; the swelling ratio was even higher for mannose, but the gel remained unchanged in the presence of galactose.

For sugar sensing, the osmotic mechanism applies only at low ionic strength, whereas changes in the crosslink density is operational at high ionic strength. Phenylboronic acid has been used for saccharides sensing in the frame of both mechanisms. Asher *et al*. synthesized acrylamide copolymers containing phenylboronic acid [69]. At low glucose concentrations, the glucose molecule cross-linked two boronate groups thus inducing gel shrinking. However, higher amounts of glucose saturated the boronate sites and induced breakage of the crosslinks, thus swelling of the gel. The degree of swelling was dependent upon glucose concentration. The hydrogel sensor was operational in physiologically relevant conditions. The method was found to be selective to glucose over other saccharides. Further modifications in the gel composition and sensing procedures allowed development of high glucose concentration responsive hydrogels [70,71], fast responsive hydrogels [72], materials for noninvasive monitoring of glucose level in tear fluids [73] and continuous monitoring in blood [74,75].

## 4. Principles of Readout Platforms Supporting Bioresponsive Hydrogels in Biosensing

Transduction schemes in biosensing can be divided in three separate categories: electrochemical, optical, and mechanical [76]. In this part, we will give an overview of the different strategies that use hydrogel for the label free sensing of bioanalytes within these three categories.

### 4.1. Electrochemical Transduction

Electrochemical sensors represent a very important category of biosensors, in particular for commercial sensors, coming from the Clark electrode. However, for the most part, hydrogels have only had a limited impact in this category. Their potential has been felt as supports for biomolecules (enzymes, antigen, DNA) to increase the loading capacity close to the electrode (basically going from a 2D to a 3D sensor [77,78]). This stems from the fact that they combine many advantages compared to other materials: they are soft and biocompatible (mostly because they are composed for the largest part of water, 95% in most cases), they can be loaded with bioactive material (drug, biomolecules, enzyme, cells) and they have been shown to stabilize the embedded bioactive material [79,80]. Thus hydrogels have been used extensively when the stability of the active part of the sensor, such as in the case of enzymes (glucose oxidase, lactate oxidase, and alcohol oxidase), is critical for the functioning of the sensor [81]. Some of these characteristics (low inflammatory response because of its mechanical properties or because of its low non specific binding of blood plasma, or through the elution of an embedded drug) make hydrogels particularly attractive for *in vivo* applications [82–84].

More recently, the focus of electrochemical transduction involving hydrogels has been towards the miniaturization of conductimetric sensors using interdigitated electrodes to probe the conductivity of pH sensitive hydrogels [85], the use of blends of conducting polymer and redox hydrogels for more rugged glucose biosensors [86], and the fabrication of hydrogel coated ISFETs (ion sensitive field effect transistors) for the sensing of nucleotides, or monosacharides [87,88], and the formation of arrays of electrochemical sensors to probe multiple analytes using small biological samples [89,90]. Also, Guiseppi-Elie and coworkers recently pursued the use of a commercial micro-disc electrode arrays (ABTECH Scientific, Inc.) to probe hydrogels; their goal was to optimize the design parameters (geometry of the electrodes, thickness, composition of the hydrogel, and enzyme loading) to optimize the signal of an implantable amperometric biosensor [91,92]. The reader is referred to a recent review on electroconductive hydrogels to get in depth information on the subject [93]. Electrochemical transduction, because of its long standing reputation, is a favorite in industrial biosensors. The wealth of information already available on electrochemical techniques and the ease of interfacing with ever more complex monitoring systems has led to commercial success especially in the glucose sensor business (e.g., Medtronic MiniMed).

In this review, we focus on more complex systems where the structure of the hydrogel itself is affected by its local environment. In so called smart hydrogels, the environment interacts with the different components of the polymeric network (covalent, electrostatic, *etc*.) in a reversible manner or not (with the reversible option being extremely valuable as it insures the reliability of the sensor in the long term). A stimulus affects the structure of the hydrogel and this change in volume, composition or mass, is transduced using different mechanisms that we will explore in the following two sections. Thus, the uptake and release of water by the responsive polymer film are accompanied by changes in weight, mechanical properties, distribution of mechanical stresses inside the film, and refractive index. These changes in the physical properties can be transformed into readable optical or electrical signals.

### 4.2. Optical Transduction (Figure 6)

One of the most common strategies used to detect a recognition event in a biological sensor is a change of fluorescence (absorption or shift in maximum). Fluorescence is the phenomenon of choice for biologists because they are familiar with relevant detection techniques, the instruments required for fluorescence detection are used widely in research laboratories and even in hospitals because of the booming business of immunoassays, and the high sensitivity makes it possible to target low concentrations of biomolecules (thus, at least in theory, biological samples can be used directly without any purification/concentration step).

Miyata and coworkers [55] were among the first ones to foresee the potential of hydrogels as part of fluorescence biosensing schemes. Specifically, they have focused on fluorescence displacement assays involving antigen-antibody interactions [55,57]. Displacement assays rely on the displacement of bound fluorescently labeled analytes with free analytes and the subsequent measurement of changes in fluorescence absorbance [94]. Such assays are very sensitive, but they also suffer from many limitations. First, the tagged analyte must be readily available. Second, the size and position of the dye on the analyte must be such that it does not affect the binding to the hydrogel recognition site. If the binding constant of the tagged analyte is different from that of the free analyte, the saturation of the binding sites by the tagged analyte may become unreliable, thus any further quantification of free analyte binding will be affected.

The protocol used for saturation must be reproducible. Also, displacement assays are very time consuming, often requiring many hours of equilibration. Finally, when the fluorescently labeled analyte is exhausted, refilling is needed unless we assume that the sensor will only be used once or for a short amount of time. For all these reasons, fluorescent displacement assays will probably remain limited to *in vitro* assaying or at least to short term sensors.

### 4.3. Detection of Refractive Index Changes Using Surface Plasmon Resonance (SPR)

The detection of refractive index changes are less sensitive than fluorescence measurements, and have the advantage of being universal. Therefore, there has been a surge recently towards the use of surface plasmons as a new scheme to detect small changes in refractive indexes close to metallic surfaces. Surface plasmons arise from the confinement of the light at the interface between a thin metal film and a dielectric medium. Surface plasmons and their resonance frequency are extremely sensitive to minute changes in the structure of thin film, as well as the dielectric properties of the medium directly in contact with this film, and this has lead to the great success of SPR in biosensing [95]. By changing the type of nanostructures at the surface of the thin metallic film as well as their periodicity, one can increase significantly the sensitivity of the device to changes in refractive index. Hydrogels are introduced to increase the robustness of the device, render it biocompatible and increase the loading capacity of active sites within the decay length of the evanescent wave. As the volume density of the hydrogel changes through the binding of the analyte and/or the change in volume of the hydrogel, the refractive index at the interface is affected. For example, we will assume that the presence of the analyte makes the gel swell, e.g., by such mechanisms presented above. As the hydrogel swells, analyte molecules as well as water molecules enter the polymeric network, thus the volume density may increase or decrease depending on the relative degree of solvation of the polymer in the shrunk state and of the polymer/analyte in the swollen state. Consequently the refractive index in the swollen state will differ from that in the shrunk state. SPR enjoys high sensitivity even when a liquid is brought in contact with the sensor but a limitation is that the detection limit increases as the size of the analyte decreases (small molecules will have a smaller impact on the refractive index than bigger ones). A development of SPR with great potential for biosensing applications is imaging SPR [96]. Using an imaging detector and varying the wavelength of the incoming laser beam while maintaining the angle of incidence constant, wavelength dependent maxima for each pixel can be determined that correspond to a local refractive index/analyte concentration. Recently, Bo Liedberg and coworkers demonstrated the use of imaging SPR to determine the optimal conditions of the hydrogel network so that the sensitivity to a given analyte is maximal [97]. This technique could be of great help for future designs of large scale arrays of sensors.

Optical wave guide spectroscopy is another kind of spectroscopy that uses the surface plasmon resonance phenomenon. A hydrogel film is formed on the thin gold film adsorbed on a LaSFN9 glass prism. The crystal is mounted in such a way that it can be rotated around an axis to vary the angle of incidence of a transverse magnetic polarized laser beam. Assuming some characteristics of the hydrogel film (thickness and refractive index), the hydrogel film can support guided light waves. At a given angle, several resonance frequencies are observed using this set up because of interferences between the guided light wave and the plasmon resonance. Using this technique, both the thickness and the refractive index of the hydrogel film can thus be calculated, contrary to the SPR that only allows for the calculation of the refractive index [98].

### 4.4. Optical Transducers Using Light Interference and Diffraction Principles

#### Interference Based Sensors

Stokke and coworkers developed a new strategy for gel swelling determination based on a Fabry Perot cavity on a fiber optic tip. A hemispherical shaped sensitive hydrogel network is grafted to the surface of an optical fiber’s tip. The incident light gets reflected at both the fiber-gel interface and the gel-surrounding liquid interface. This creates a pattern of interferences whose phase depends on the optical path length within the hemispherical hydrogel material. With a change in the degree of swelling of the hydrogel, the spacing between the interface gel-fiber and the interface gel-surrounding solution varies so that the phase of the interferences produced by the interacting reflected beams is affected. The authors have achieved very high resolution (2 nm in displacement; revealing that the technique is very sensitive to small changes in analyte concentration) and sampling frequency (1Hz; which allows for swelling kinetic measurements) using this technique[23,99]. They have demonstrated the use of such strategy to measure glucose concentration [74,75] as well as to detect the concentration and sequence of short nucleotides [51]. In order for this strategy to work, a difference in refractive index must exist at the gel-surrounding solution interface. Multiplexing involving the use of multiple fibers, each with different gel, designs can expand the detectable concentration range of the analyte.

#### Bragg Diffraction Based Sensors

For more than 15 years, Asher and coworkers [33] have been developing a rather unique strategy to make hydrogel based sensors. The strategy is based on embedding a crystalline colloidal array within a biosensitive (Lead, [35], Glucose [71], creatinine [41] or ammonia [100]) hydrogel matrix. As the crystalline colloidal array is formed on a substrate, it adopts a face centered cubic structure that diffracts light according to Braggs diffraction law [101]. As the hydrogel shrinks/swells according to the concentration of the analyte, the spacing/arrangement between the colloids changes, thus affecting light diffraction through the hydrogel film. Shifts in the wavelength of the diffraction maximum are recorded and correlated with changes in the concentration of the analyte through the use of a calibration. For example, when phenylboronic acid groups are introduced in the hydrogel network, the crystalline colloidal crystal becomes sensitive to the concentration of glucose in aqueous solutions. Asher demonstrated the capability of this system to diffract light in a narrow wavelength band in the visible region. The small half peak width ensures that the sensor is sensitive to small changes in glucose concentration. The fact that the wavelength range is in the visible is critical for this specific application because it is well known that the main obstacle to continuous monitoring of glucose levels (which has been shown to have a direct impact on the life expectancy of diabetes patients) is the compliance of the patient to the routine testing, so the sensor must be simple and pain free to use. Asher envisions his photonic crystal as a part of a contact lens because the level of glucose in tear fluid is correlated with that in blood sugar. A simple observation of a color change in mirror and comparison to a color scale calibrated in blood sugar level would greatly benefit diabetic patients’ health [71,73].

Several other groups have subsequently developed strategies based on similar crystalline arrays. Braun’s group used the polymeric colloidal crystal array as templates. Once formed in a similar manner as Asher’s, the colloidal portion is dissolved to form a hollow structure that diffracts light [20]. Li and coworkers combined this new strategy with molecular imprinting where an unbound analyte is added to the hydrogel before gelation so that upon successive washing of the inverse opal structure, voids are formed shaped as the analyte to be targeted by the sensor. They were able to successfully achieve chiral recognition of l- and d-Dopa [59] as well as determine cholic acid (gastric acid) [26] and performance enhancing drugs [102] using this strategy.

Collectively, strategies that use polymerized colloidal crystal arrays are very valuable because of their high sensitivity and specificity, quick response, and reliable reproducibility. While Asher’s crystalline colloidal crystal arrays offer very high sensitivity because the formation of large domains of single crystal leads to intense and narrow Bragg diffraction band, the hydrogel system needs to be formed without disturbing the already organized array. This limits the variety of analytes that can be targeted especially for charged hydrogels. Another disadvantage of Asher’s system is the rather large diffusion barrier for analytes to diffuse throughout the crystal; to overcome this limitation high water content is favored, but this can inversely affect the mechanical stability of the array. In Braun’s inverse opals [20], the removal of the original crystal introduces defects in the crystal structure but the technique offers more flexibility in the components (functional groups, charges) that can be introduced in the hydrogel system and the interconnection between cavities allow for rapid diffusion of the analyte in the network hence increasing the sampling rate of the sensor. Maintaining the mechanical integrity of the inverse opals is a crucial issue but recent advances in hydrogels toughening may help alleviate this specific issue.

An interesting application of diffraction in biosensing is the holographic diffraction grating created by Lowe and coworkers [103]. Lines of silver nanoparticles regularly spaced out are produced *in situ* within a hydrogel matrix using a photosensitization process. The grating spacing is dependent on the swelling of the hydrogel thus acting as a wavelength filter for the light reflected from it. The major practical hurdle for the use of this strategy lies on the fabrication of the nanoparticle array itself (contrary to the polymerized colloidal crystal arrays) since it requires the use of a frequency-doubled Nd-YAG laser. These holographic sensors were applied to fabricate sensors for penicillin and urea [104], glucose [105], and recently they formed enzyme inhibition assays [106].

#### Microlens Sensors

Recently, hydrogels have been integrated in microlenses designs for biosensor application. In the system described by Lyon and coworkers [107,108], microlenses are formed by direct absorbtion of microgel on a substrate surface; upon absorption, the microgel adopts a lens shape. Changes in the hydrogel swelling affect the shape of the lens and, hence, its focal length. There are three major advantages to this technique. First, the quantification of absorption of analyte can be derived from a simple observation using a brightfield optical microscope. Second, the sensing scheme is totally autonomous (no power supply) and third, the formation of complex arrays of microlenses with automatic readout is conceivable. Jiang and coworkers [109] adopted a slightly different lens design (with the hydrogel lens encapsulated within a micromachined device and submerged under a film of oil) that could potentially be integrated in a larger sensing scheme and further miniaturized. There again, the sensing occurs through displacement assays (notoriously slow) so the sampling rate (which depends on the diffusion of the analyte, and consequently on the hydrogel volume) is critical.

The use of optical transducers, no matter how elegant, carries the disadvantage of having to be interfaced with a light source and a light detector, which require an electrical circuit, thus increasing the footprint of the whole sensor [110]. This is an issue of cost as well as complexity of design; as the number of elements is increased, the reliability of the overall sensing scheme may become problematic and the number of competencies needed to troubleshoot such a design makes it unpractical for mainstream applications. Specifically, this is a concern when looking to make an *in vivo* sensor; continuous monitoring of a biological event *in vivo* (blood sugar being the best example) is the holy grail of the sensing industry and it is unclear whether optical transducer schemes will ever reach the degree of integration that MEMS type transducers already have. Indeed, MEMS type devices have been produced for many years in different fields; their small size, the reliability of the designs, the low power usage, and the ease of their integration in an electrical circuit for the readout platform makes them prime choices for *in vivo* applications. Conversely, optical transducers have found their niche market in the fabrication of laboratory based instrumentation.

### 4.5. Mechanical Transduction (Figure 7)

The emergence of mechanical transduction is rather recent compared with the previous two categories of transduction. Microfabrication technologies derived from the microelectronics industry has played a central role in the miniaturization of biosensors (required for implantation as well as for the integration of multianalyte biosensors within the same sensing chip) while keeping the fabrication process reliable, and keeping the costs down.

#### Pressure Sensors

**Figure 8.**
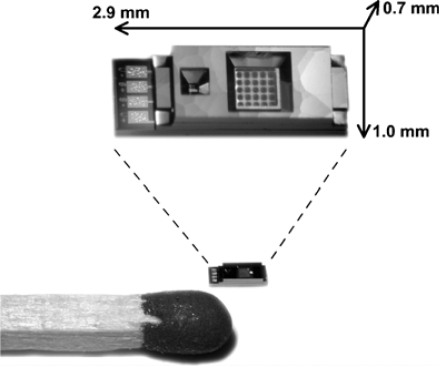
Microfabricated sensor based on the swelling of a pH sensitive hydrogel and used as a carbon dioxide sensor [115]. © 2005 Springer Science+Business Media, Inc. Reproduced with the permission from the publisher.

Most studies involving environmentally sensitive hydrogels have focused on gels being in an equilibrium state at constant pressure. Under such conditions, the hydrogel polymeric network can expand freely to accommodate an imbalance in osmotic pressure between the inside and the outside of the gel and reach a new equilibrium state. However, if the gel is encapsulated inside a solid structure (with a semi-impermeable membrane allowing its interaction with the environment), the polymer network will exert a pressure over its casing. Magda, Solzbacher [111,112] and Van den Berg [113] have demonstrated different systems where one of the sides of the casing is a piezoresistive pressure sensor. As the hydrogel presses on the membrane of the pressure sensor, the latter bends slightly, thus affecting a piezoresistive element based on a Wheatstone bridge and the electrical signal can be recorded instantaneously. The increase in volume created during the bending of the pressure sensor membrane is extremely small compared with the size of the cavity such that one can consider that the sensing event happens at constant volume. Such a sensing scheme has the advantage to be completely label free so it can potentially be applied to any number of hydrogels without any further modification. Magda and Solzbacher [114] showed the capability to relate the osmotic pressure produced by monosaccharide sensitive hydrogel with the concentration of sugars. Van Den Berg [115] used a two step system where CO_2_ vapor is brought in contact with a reservoir electrolyte (through a PDMS membrane) which is itself in contact with an enclosed pH sensitive hydrogel (through a microfabricated semi-permeable membrane). While the first two authors used a commercially available millimeter size pressure sensor, Van den Berg integrated an unpackaged pressure sensor in a smaller, more complex design, demonstrating the potential for its commercial application (Figure 8).

### 4.6. Capacitive Sensors

Capacitive biosensors are uncommon, and several inherent properties of these sensors may explain this. They require multiple process steps for their fabrication which makes them relatively expensive compared to some of the other sensors described in this paper. Instruments to measure capacitance directly are rather insensitive. They suffer from reliability issues when scaled down below a certain size especially in liquids. However, they can be integrated with an inductance to form a LC circuit whose resonance frequency can be measured very precisely and wirelessly [116,117]. This is especially valuable for the continuous monitoring of blood sugar as demonstrated by Siegel and Ziaie [118].

### 4.7. Cantilever Based Sensors

Recently, there has been a considerable amount of research in the modification of silicon cantilevers with bio-sensitive layers for sensing applications [119–121]. A hydrogel film is bound to the top or bottom of the cantilever [122,123]; as the hydrogel swells/shrink, the lateral stress it exerts on the thin silicon beam provokes its bending [124]. The bending can be recorded either by a change in position of a laser beam on a four quadrant photosensor after reflecting off the tip of the cantilever (if the hydrogel is on top of the beam, a patch needs to be left unfunctionalized) or using a piezoresistive element integrated in the cantilever. The amount of deformation of the cantilever beam can be correlated to the degree of swelling of the hydrogel film; because this process is completely reversible, an empirical calibration curve can easily be determined. Such capability, the ease by which different types of hydrogels can be adapted to the same design, and the possibility to functionalize specific cantilever within an array using photolithography definitely makes this transduction mechanism very promising.

### 4.8. Microgravimetric Sensors

The simplest type of microgravimetric sensor is the Quartz Crystal Microbalance (QCM). In a QCM, a piezoelectric element is driven using an alternating current such that it vibrates at a defined frequency, then the frequency is scanned while recording the amplitude of oscillations to find the resonance frequency. QCM resonators are sensitive to two distinct parameters. First, the weight of the material (so that smaller molecules will have a higher detection limit than bigger ones), which manifests itself as a shift in the resonance frequency, is the energy storage term. Second, the viscoelasticity of the hydrogel thin film, which affects the rate of decay of the oscillations, is the energy dissipation term [125]. The advantages are similar to those for SPR: impedance measurements are universal and frequency can be measured in a very precise manner. The hydrogel provides the system with binding specificity and increased capacity with respect to a layer of binding sites on the piezoelectric device. QCM has been applied to fabricate sensors for cell detection [126], DNA [127], sepsis-related biomarkers [128], proteases [129], and glucose [130]. However, QCM can be difficult to implement in real time and with low volume sensor systems.

## 5. Conclusions

While it has been clearly demonstrated that hydrogels can play a role in increasing functionality within biosensing, either by enhancing loading capacity compared to that accessible to surfaces or by including topological designs that transform biospecific recognition events into signals that can be readily processed, only limited quantitative optimization of the hydrogel systems designed has been realized. Attempts of quantification have been made especially for the hydrogel matrices of the colloid crystalline arrays [71]. However, a recent report on the effect of the size of the integrated colloids on the mechanical properties [131] indicates that such quantitative description requires a calibration between the theoretical parameters and the experimental data. Nevertheless, further research is needed to better understand the quantitative relationship between the hydrogel parameters selected and the output signal of specific readout platforms. Future research in this field should also consider relevant properties such as response time, analyte transport, and optimization of the material in view of practical applications.

An additional challenge that still largely remains to be tackled is the development of more efficient techniques to transduce changes in volume or mass of a hydrogel system into a real life sensing device. Such integration requires the collaboration of scientists and engineers across multiple fields of research. Most certainly, academics are better positioned to do this than industrial researchers. Such issues as reliability, robustness, ease of fabrication, and overall cost compared with other established technologies will have to be addressed to the same extent as pure performance of the sensors before hydrogel sensors can make it to market.

## Figures and Tables

**Figure 6.**
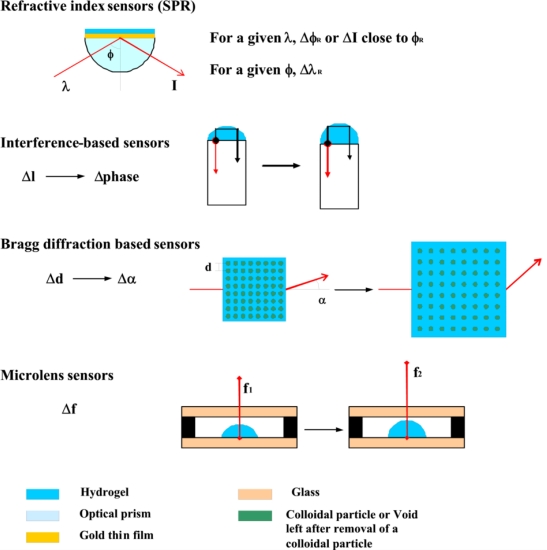
Schematics of the principal optical transduction schemes. λ: wavelength, λ_R_: resonance wavelength, I: intensity, ϕ_R_: resonance angle, l: optical path, d: lattice spacing, α: diffraction angle, f: focal length.

**Figure 7.**
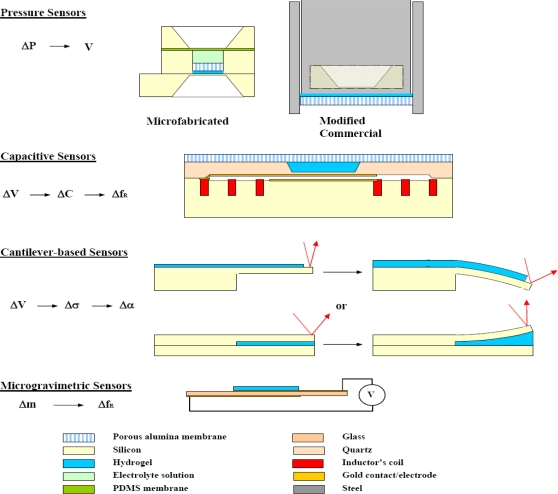
Schematics of the principal mechanical transduction schemes. P: pressure, V: potential difference, ΔV: volume change, C: capacitance, f_R_: resonance frequency, σ: mechanical stress, α: angle, m: mass, f: frequency.
